# Cancer Occurrence as the Upcoming Complications of COVID-19

**DOI:** 10.3389/fmolb.2021.813175

**Published:** 2022-01-28

**Authors:** Ilnaz Rahimmanesh, Laleh Shariati, Nasim Dana, Yasaman Esmaeili, Golnaz Vaseghi, Shaghayegh Haghjooy Javanmard

**Affiliations:** ^1^ Applied Physiology Research Center, Isfahan Cardiovascular Research Institute, Isfahan University of Medical Sciences, Isfahan, Iran; ^2^ Department of Biomaterials, Nanotechnology and Tissue Engineering, School of Advanced Technologies in Medicine, Isfahan University of Medical Sciences, Isfahan, Iran; ^3^ Cancer Prevention Research Center, Isfahan University of Medical Sciences, Isfahan, Iran; ^4^ Biosensor Research Center, School of Advanced Technologies in Medicine, Isfahan University of Medical Sciences, Isfahan, Iran; ^5^ Isfahan Cardiovascular Research Center, Cardiovascular Research Institute, Isfahan University of Medical Sciences, Isfahan, Iran

**Keywords:** COVID-19 sequelae, SARS-CoV-2, cancer, long COVID-19, immune homeostasis

## Abstract

Previous studies suggested that patients with comorbidities including cancer had a higher risk of mortality or developing more severe forms of COVID-19. The interaction of cancer and COVID-19 is unrecognized and potential long-term effects of COVID-19 on cancer outcome remain to be explored. Furthermore, whether COVID‐19 increases the risk of cancer in those without previous history of malignancies, has not yet been studied. Cancer progression, recurrence and metastasis depend on the complex interaction between the tumor and the host inflammatory response. Extreme proinflammatory cytokine release (cytokine storm) and multi‐organ failure are hallmarks of severe COVID‐19. Besides impaired T-Cell response, elevated levels of cytokines, growth factors and also chemokines in the plasma of patients in the acute phase of COVID-19 as well as tissue damage and chronic low‐grade inflammation in “long COVID‐19” syndrome may facilitate cancer progression and recurrence. Following a systemic inflammatory response syndrome, some counterbalancing compensatory anti-inflammatory mechanisms will be activated to restore immune homeostasis. On the other hand, there remains the possibility of the integration of SARS- CoV-2 into the host genome, which potentially may cause cancer. These mechanisms have also been shown to be implicated in both tumorigenesis and metastasis. In this review, we are going to focus on potential mechanisms and the molecular interplay, which connect COVID-19, inflammation, and immune-mediated tumor progression that may propose a framework to understand the possible role of COVID-19 infection in tumorgenesis and cancer progression.

## Introduction

Over the previous decades, viral infections have posed significant challenges for cancer management. Several oncogenic viruses are known to cause cancer. However, there is no evidence linking between cancer subtypes and Severe Acute Respiratory Syndrome-Coronavirus-2 (SARS-CoV-2) infections in clinical and pre-clinical studies. Cancer patients are more susceptible to SARS-CoV-2 infection with possible poor prognosis than normal population due to their systemic immunosuppressive state caused by the cancer and anticancer treatments, such as active chemotherapy. The perpetually growing numbers of COVID-19 infections and the increase in numbers of diagnosed and undiagnosed cancer patients have warranted apprehending the interrelationship between COVID-19 and cancer. COVID-19 is concomitant with activation of oncogenic pathways, including The Janus kinase signal transducer and activator of transcription)JAK-STAT (, mitogen-activated protein kinase (MAPK), and Nuclear factor kappa B (NF-κB), which potentially can increase the risk of cancer (Li et al., 2020a).

On the other hand, COVID-19 and cancer look alike in several ways such as inappropriate T-cell responses. Another similarity is antigenic stimulation caused by damage-associated molecular pattern (DAMP) and pathogen-associated molecular pattern (PAMP) molecules occurs in both cancer and infectious disease (Hotchkiss and Moldawer, 2014). DAMP and PAMP figure out inflammation, leading to the release of various cytokines, increased levels of reactive oxygen and nitrogen species, tissues damage, and apoptosis. Moreover, hypoxia as well as hypoxic microenvironment secondary to inflammation or virus‐induced angiotensin‐converting enzyme 2 depletion provoke oxidative stress and probable malignant transformation (Saini and Aneja, 2021). In addition, the hypoxic microenvironment results in lysyl oxidase (LOX) production, which increases tumor cells invasion and facilitates migration and metastasis (Ye et al., 2020).

Immune responses in acute phase of COVID-19 patients, called cytokine storm are arranged by proinflammatory cytokines, which are also recognized to promote tumorigenesis (Del Valle et al., 2020).

Furthermore, the consecutive low-grade inflammation seen in COVID-19 patients after acute phase may result in a constant cycle of inflammation-induced organ injury and injury-induced inflammation. Although, cancer development is never the consequence of an insulated event; it could be hypothesized that COVID-19 may predispose the human to cancer development and accelerate cancer progression and metastasis. In this review, we discuss the pathways and hypotheses that may expose patients with COVID-19 to cancer in the future.

## Epidemiology and Clinical Characteristics of COVID-19

The recent coronaviruses epidemics have rapidly spread with irreparable consequences (Guan et al., 2020; Sohrabi et al., 2020). As of 28 October 2021, the World Health Organization (WHO) reported a total of 245M confirmed cases with 4.97M deaths, making it as one of the deadliest crisis in history (Dikid et al., 2020).

Coronaviruses can cause multiple organs infection especially respiratory tract infection. The clinical signs and symptoms are typically including fever, dry cough, muscle pain, diarrhea, and breathing difficulties (Abdel-Moneim and Hosni, 2021). Sever cases of COVID-19 represent acute respiratory distress syndrome (ARDS), sepsis and septic shock, multiorgan failure leading to death (Zafer et al., 2021). Indeed, in these cases, viruses can evade the immune system and spread to other organs, such as cardiovascular system, gastrointestinal system, central nervous system, liver and kidney, where they cause a variety of serious diseases (Bajaj et al., 2021; Soltani et al., 2021). Apart from the consequences created by the coronaviruses, underlying medical conditions such as cancer, cardiovascular diseases, renal diseases, and type I/II diabetes, increase the risk for severe COVID-19 infection (Haybar et al., 2020).

## Structure, Life Cycle and Pathogenesis of SARS-CoV-2

SARS-CoV-2 is a positively-sensed, and single-stranded RNA-enveloped virus with spherical capsids (120–160 nm) (Hoffmann et al., 2020; Nakagawa and Makino, 2021). The coronaviruse genome (GenBank no. MN908947) is about 26.4–31.7 kb long, making the largest among RNA viruses encoding 9,860 amino acids (Woo et al., 2010). Both structural and nonstructural proteins are found in gene fragments. Non-structural proteins such as 3-chymotrypsin-like protease, papain-like protease, and RNA-dependent RNA polymerase that are encoded by open reading frame (ORF) region, while structural proteins such as spike protein (S), envelope protein (E), membrane protein (M) and nucleocapsid protein (N).

**FIGURE 1:**
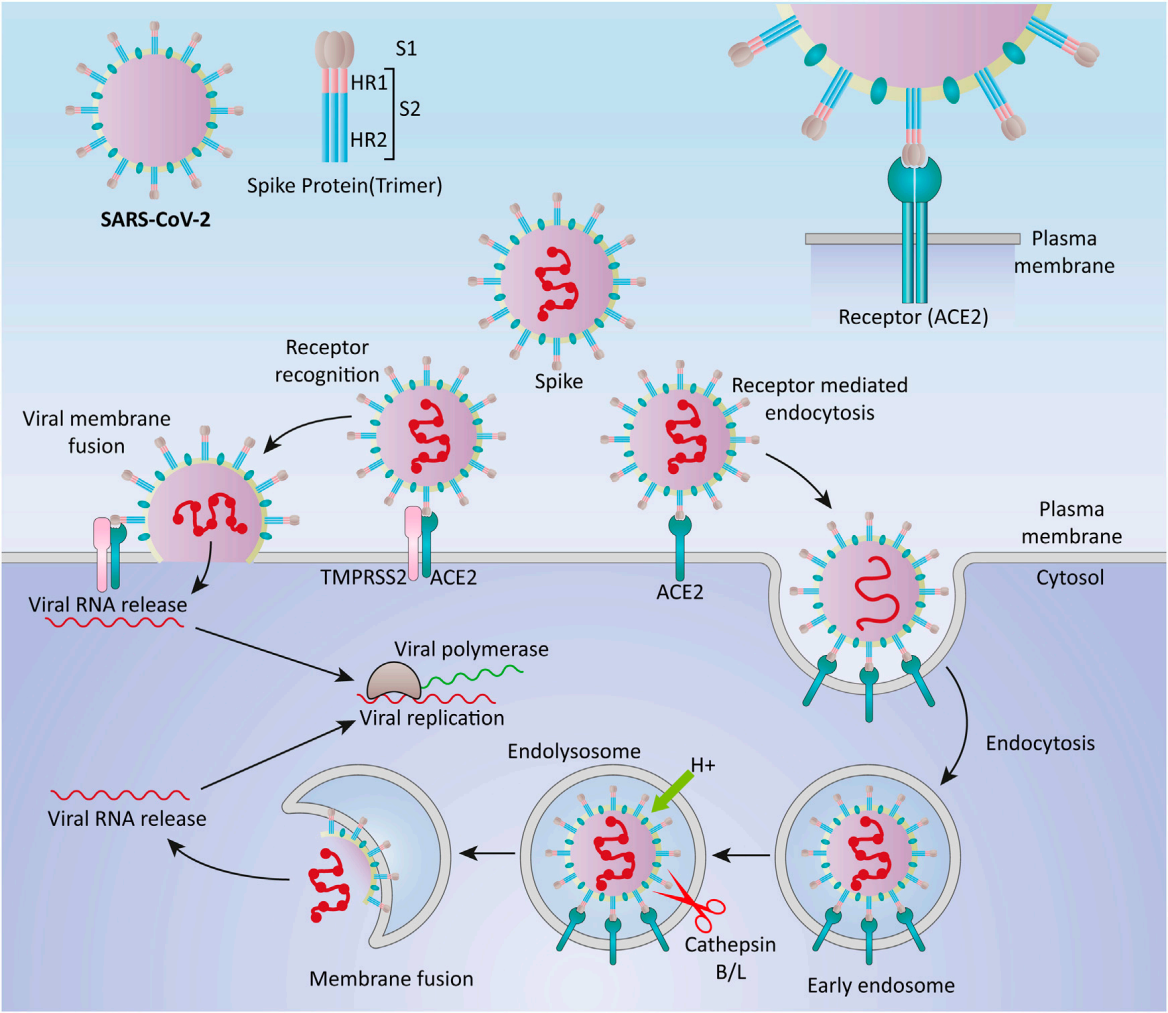
Cell entry mechanisms of SARS-CoV-2. Coronaviruses are believed to enter the host cells *via* two main routes, through direct delivery of the genome into the cytosol *via* fusion with the host cell membrane, and through endocytosis.

SARS-CoV-2 surface is covered by the S protein, a large glycosylated transmembrane protein (1,160–1,400 aa) that binds to the host cell receptor angiotensin-converting enzyme 2 (ACE2) and mediates viral cell entrance (de Wit et al., 2016; Wu et al., 2020). Since the pathogenesis of coronaviruses has not been completely understood, the precise molecular mechanism of entry of viruses into cells remains unclear (Glebov, 2020). Coronaviruses are thought to enter the host cells by two main routes: direct genome into the cytosol *via* fusion with the host cell membrane, and endocytosis (Pelkmans and Helenius, 2003) (Figure 1). Typically, when the S protein attaches to the receptor, proteases on the host cell membrane, such as transmembrane protease serine 2 (TMPRSS2) and airway trypsin-like protease TMPRSS11D, promote virus entry into the cell by activating the S protein. The viral RNA genome is translated into two polyproteins and structural proteins in the cytoplasm once the virus enters the cell, facilitating the construction of virus progeny (Perlman and Netland, 2009). Replication and transcription of the viral RNA genome occur through protein cleavage with continuation/discontinuation of RNA synthesis that is mediated by a replicase-transcriptase complex. Eventually, structural proteins are synthesized, assembled, and packaged in the endoplasmic reticulum-Golgi intermediate compartment of the host cell (Li et al., 2020b).

Furthermore, it has been proposed that SARS-CoV-2 entered cells through clathrin-mediated endocytosis (Bayati et al., 2020). SARS-CoV-2, like SARS-CoV and MERS-CoV, may use numerous pathways to successfully enter the cytosol of the host cell (Hartenian et al., 2020; Yang and Shen, 2020).

## COVID-19 Infection and the Immune System Responses

**FIGURE 2:**
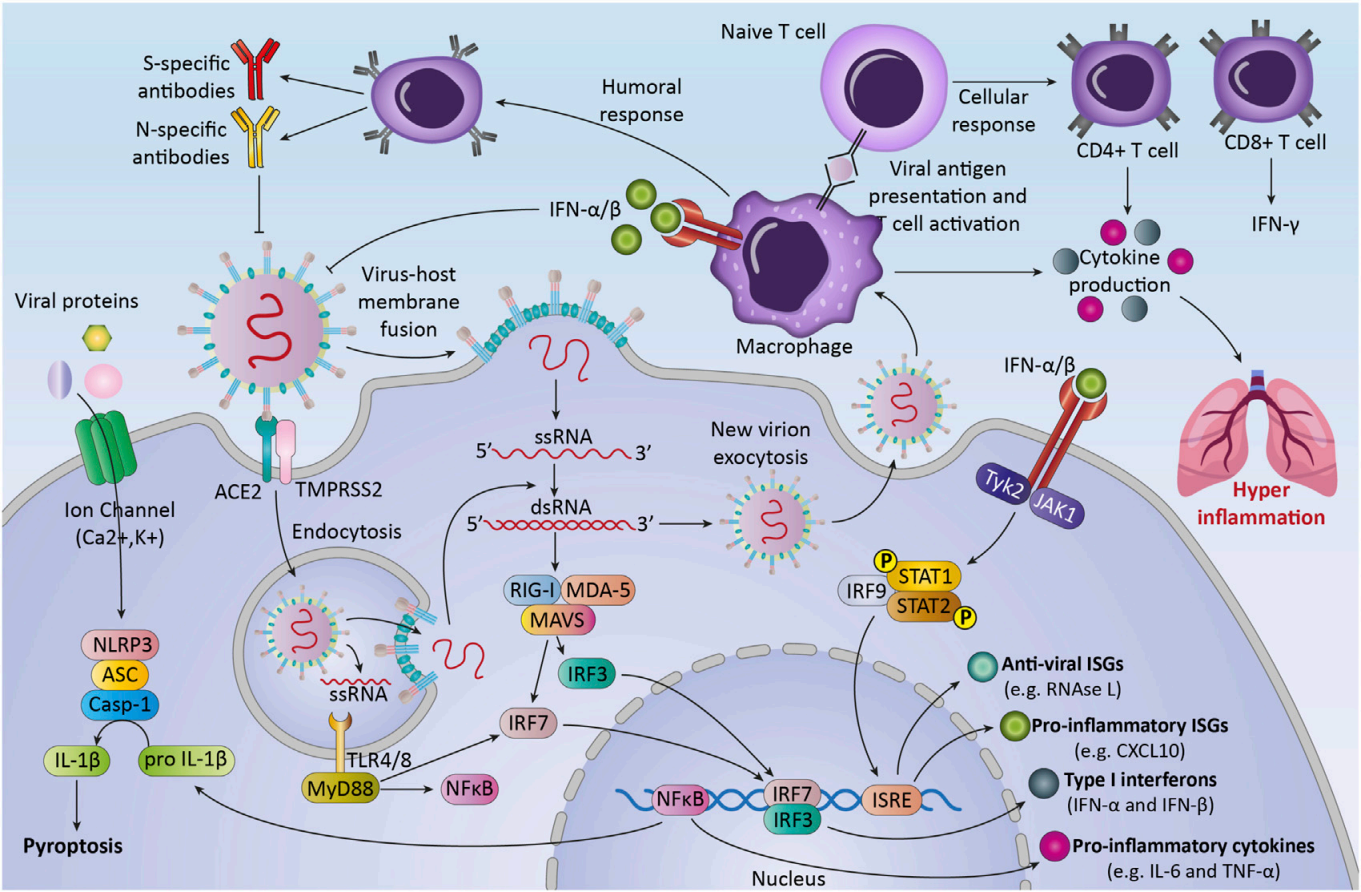
The immune response against SARS-CoV-2.The first line of host defense against SARS-CoV2, innate immunity, is the response against the virus by recognizing Pattern associated molecular patterns (PAMPs) through transmembrane or intracellular pattern recognition receptors (PRRs). Recognition of viral components leads to the activation of immune cells and transcription factors that lead to the production of different cytokines, chemokines, and anti-viral proteins. These processes can lead to activation of adaptive immune response that consists of three major cell types (B cells, CD4^+^ T cells, and CD8^+^ T cells). After pathogen elimination, adaptive immunity regulates innate immunity to avoid unnecessary host cell damage. Unbalanced response and immune system overactivation can cause collateral damage to host tissues and exacerbate disease severity.

The immune system responses to SARS-CoV-2 are considered to be critical in controlling the pathogenicity and clinical symptoms of the patient (Yang et al., 2020). After virus infection, the immune system uses multiple mechanisms for recognition and defends against viruses (Koch et al., 2013). The body uses both the immune responses (innate and adaptive immunity), to eliminate viral infection (Ye et al., 2021) (Figure 2). During the first hours and days, the innate immune response to viruses begins with innate immune cells, like phagocytic cells (neutrophils, macrophages) and dendritic cells (Zhou and Ye, 2021). These cells express different types of Pattern-recognition receptors (PRRs) to recognize DAMPs and PAMPs (Li and Wu, 2021). In COVID-19 patients, NK cells can kill virus-infected cells by releasing cytotoxic granules or through participating in antibody-dependent cellular cytotoxicity and producing different cytokines and chemokines (Ma et al., 2021).

After the fusion of the virus into the cells, viral genome starts replication and transcription (Astuti and Ysrafil, 2020). Then immune system recognized RNA viruses by cytosolic and endosomal RNA receptors, such as RIG-I-like receptors and toll-like receptors (TLR3, TLR7, and TLR8) (Hosseini et al., 2020). Within hours, RNA virus recognition by these receptors can activate some of the transcription factors like nuclear factor kappa-light-chain- enhancer of activated B cells (NF-κB), to produce pro-inflammatory cytokines and chemokines and activation of immune cells (Kircheis et al., 2020). The result of these processes is the production of interferons -λ (type III IFN) and type I IFN, pro-inflammatory cytokines (for example, interleukin-6, interleukin-18, and interleukin-1) and some chemokines such as chemokine (C-C motif) ligand 2 (CCL2) and chemokine (C-C motif) ligand 7 (CCL7) by immune cells (Stetson and Medzhitov, 2006).

Moreover, the infected cells may undergo the process of inflammatory cell death (pyroptosis) and release DAMPs, such as viral nucleic acids and oligomers (Yap et al., 2020).

The second line of defense, the adaptive immune response, happens several days later after the innate immune response occurs. T cells and B cells, two classes of lymphocytes, carry out the adaptive immune response against COVID-19. In the majority of infected patients, B cells at first produce IgM and IgA antibodies and then IgG, that is measurable between weeks two and five after infection (Emmerich et al., 2021). Various structural proteins of the virus can be targeted with these antibodies (Pan et al., 2021).

The other adaptive immune cells, CD8^+^ T cells, directly kill cells that are infected with the virus and stop virus spreading further (de Candia et al., 2021). The other type of T cells, CD4^+^ T cells, produce different types of cytokines and chemokines that influence the function of B and T cells, and other immune cells such as macrophages (Chen et al., 2010).

After pathogen elimination, adaptive immunity regulates innate immunity to avoid unnecessary host cell damage (Zhou et al., 2020). Notably, while a highly organized immune response to viral infection is important, an unbalanced response and immune system overactivation inadvertently targeting healthy cells and lead to host tissues damage (Catanzaro et al., 2020). The imbalance of the immune system in fighting against SARS-CoV-2 can induce inflammation, lymphopenia, and cytokine storm in patients and follow that septic shock, acute lung injury, ARDS, and multi-organs failure may occur, that increases patient’s mortality rate (Jamal et al., 2021). Early studies indicated that COVID-19 patients have higher neutrophil and lower lymphocyte counts in blood compared with healthy controls (Eslamijouybari et al., 2020). Also, CD8^+^ lymphocytes and NK cells show an exhausted phenotype and significant reduction in severe COVID-19 patients compared with patients with mild SARS-CoV-2 -infection and healthy controls (Zheng et al., 2020). These changes can disrupt the immune system and the antiviral effects of immune cells. On the other hand, cytokine storm can occur as a result of hyperactivity of infected macrophages and other immune cells that cause the production of various inflammatory cytokines (Zheng et al., 2020).

Increasing NF-κB has an important effect on cytokine storms created by an acute respiratory RNA virus. Activation of TLRs by recognition of virus PAMPs leads to downstream signaling pathways like NF-κB and interferon regulatory factor 3 (IRF3) (Hariharan et al., 2021). NF-κB plays key roles in regulating different cellular functions, such as cell growth and cell death, immune responses, and inflammation (Barnabei et al., 2021). It has been shown that COVID-19 is able to activate TLR4-mediated NF-κB signaling (Kircheis et al., 2020). Overactivation of NF-κB targets genes in the context of inflammation and affect the activation of adhesion molecules and inflammatory cells that all can cause or worsen lung disease and is a sign of chronic inflammation (Liu et al., 2017). A growing body of evidence suggests that infection and chronic inflammation as a risk factor can lead to cancer and develop some other diseases (Rossi et al., 2021). Therefore, further investigation about the interaction among the immune system and coronavirus can be effective to reduce its adverse consequences.

## Does Immune Dysregulation Predisposes Individual to Cancer

### IL-6/JAK/STAT Signaling Pathway

Infection with SARS-CoV-2 causes innate immune responses, which result in a diverse set of immune mediators and the activation of some signaling pathways, including Janus kinase/signal transducer and activator of transcription (JAK/STAT), nuclear factor kappa B (NF-κB), interferon response factor 3 (IRF3), and IRF7. The signaling cascade that results from these pathways in infected cells increases the production of pro-inflammatory cytokines (Li et al., 2020c; Prompetchara et al., 2020).

In COVID-19 related cytokine storm syndrome (COVID-CSS), different inflammatory cytokines such as Interleukin-1 (IL-1), Interleukin-10 (IL-10), and tumor necrosis factor (TNF)-α are elevated about 2-100 fold above normative values. In some patients with COVID-19, IL-6 levels rise more than 1,000 fold above normal range (Blanco-Melo et al., 2020; Herold et al., 2020; Laing et al., 2020; Al-Baadani et al., 2021). IL-6 signals through both classic and trans-signaling pathways, activates JAK-STAT3 signaling. The IL-6/JAK/STAT3 pathway is also abnormally activated in many types of cancer, which is generally associated with a poor clinical prognosis (Johnson et al., 2018). In the tumor microenvironment, IL-6/JAK/STAT3 signaling pathway plays a pivotal role in regulating the growth, survival, invasiveness, metastasis, and development of many cancers. In addition, the IL-6/JAK/STAT3 acts as a key component in suppressing the antitumor immune response (Bromberg and Wang, 2009; Kumari et al., 2016). Previous research identified the role of IL-6 as a driver of tumorigenesis and anti-apoptosis signaling and a vital biomarker of cancer diagnosis, risk, and prognosis (Vargas and Harris, 2016). Also, IL-6 enhanced the metastasis rate in breast cancer through the positive effect on tumor stem cell self-renewal and epithelial-to-mesenchymal transition (EMT) process (Bharti et al., 2016; Xiao et al., 2017).

STAT3 hyperactivation in tumor cells can occur as a result of increased IL-6 levels in the serum and/or the tumor microenvironment, or as a result of loss-of-function mutations affecting STAT3 negative regulators. IL-6 is produced by different cell types including stromal cells, tumor-infiltrating immune cells, and the tumor cells (Nozawa et al., 2006; Walter et al., 2009; Nagasaki et al., 2014). IL-6 directly stimulates the expression of STAT3 downstream targets in tumor cells. STAT3 signaling controls the expression of genes involved in tumor growth, survival, invasion, and angiogenesis. The ability of STAT3 to promote IL6 gene expression is *via* binding to the IL6 promoter, which results in a positive autocrine feedback loop (Chang et al., 2013). STAT3 also promotes angiogenesis, invasiveness, and/or metastasis, as well as immunosuppression (Teng et al., 2014; Huynh et al., 2017).

In addition to direct effects on tumor cells, IL-6 and JAK/STAT3 signaling can have a profound effect on tumor-infiltrating immune cells. STAT3 is often hyperactivated in tumor-infiltrating immune cells and negatively regulates natural killer (NK) cells, neutrophils, effector T cells, and dendritic cells (DCs). Taken together, these data suggest that, STAT3 activation in immune cells probably leads to down-modulation of antitumor immunity response (Harris et al., 2007; Yu et al., 2007; Herrmann et al., 2010; Iwata-Kajihara et al., 2011). Collectively, IL-6/JAK/STAT signaling pathway plays a fundamental role in tumorgenesis and immunosuppressive tumor microenvironment (Kortylewski et al., 2005; Yu et al., 2009).

### NFκB Pathway

The hyper-activation of NF-κB pathway has been implicated in the pathogenesis of the severe/critical COVID-19 phenotype (Hirano and Murakami, 2020). The excessive NF-κB activation subsequent to viral proteins detection *via* the innate immune system, probably has a causative role in covid-19 cytokine storm, extrapulmonary manifestations of COVID-19, and fatality rate (Liao et al., 2005; Oeckinghaus and Ghosh, 2009; DeDiego et al., 2014). This ‘rapid acting’ primary transcription factor seems to exert its effects by stimulating the gene expression of a wide variety of cytokines, adhesion molecules, chemokines, acute phase proteins, and inducible effector enzymes (Liang et al., 2004; Zhang et al., 2017).

In a similar way, NFκB is an important signaling pathway involved in the pathogenesis of tumors, and the potential role of excessive activation of this signaling pathway in oncogenesis has been confirmed. In various tumor types, intervention in this signaling pathway for targeted cancer therapy has been reported. NFκB signaling is involved in cell biogenic activities such as inflammation, cellular immunity, and stress (Salazar et al., 2014; Sau et al., 2016; Song et al., 2017). In lymphatic cancer, breast cancer, and colon cancer, the excessive activation of the NFκB-signaling pathway leads to uncontrolled and abnormal cell proliferation, differentiation, and apoptosis, as well as metastasis, and treatment resistance. The NFκB pathway is often altered in both solid and hematopoietic malignancies, promoting tumor-cell proliferation and survival (Hayden and Ghosh, 2008; Walther et al., 2015; Forlani et al., 2016; Sau et al., 2016). Therefore, the activation of NFκB pathway, as a shared pathway between COVID-19 and some cancers, plays an important role in disease progression.

### IFN-I Signaling

IFNs are members of a large family of cytokines that are currently divided into three groups (type I, II, and III IFNs) (Negishi et al., 2018). Type I IFNs are involved in the development of innate and adaptive immune responses against both cancer and infectious diseases (Bonjardim, 2005). Similar to IL-6, IFN-Is signaling activates the JAK/STAT pathway (Schindler et al., 2007). Recent studies have demonstrated that IFN signaling could be a key mechanism in tumor proliferation. Inhibition of IFN pathway induction and dysregulated IFN signaling promote tumor cell senescence and death (Sangfelt et al., 1999; Fuertes et al., 2011; Schreiber et al., 2011). Type I interferons are crucial for restricting responses during the early stage of SARS-CoV-2 infection, however, IFN-I signaling was dampened in patients with severe COVID-19 (Blanco-Melo et al., 2020), and low level of IFNs along with an increase in IL-6, have been detected in the peripheral blood samples or lungs of patients (Hadjadj et al., 2020). These data illustrate the possible role of impaired INF-I signaling induced by SARS-CoV-2 infection, in inefficient antitumor response, which leads to tumor progression. On the other hand, lymphopenia, functional exhaustion, and impaired cellular immunity responses are associated with IFN production suppression. This notion is supported by evidence, which highlights the role of IFNs in effective T cell proliferation and survival (Marrack et al., 1999). The continued production of pro-inflammatory mediators due to viral persistence has a negative effect on natural killer cells and CD8^+^ T-cell activation in the case of COVID-19 infection. The CD8^+^ T-cell population undergoes quantitative and qualitative changes. Decreased in NK cells, CD4^+^ T cells, and CD8^+^ T cell population and impaired activation phenotypes are frequently observed, particularly in the severe form of COVID-19 disease (Ai et al., 2020; Pedersen and Ho, 2020). In COVID-19 patients specific cell population, the cytotoxic CD3^−^CD56dimCD16+, was significantly reduced, and more importantly, elevated expression of regulatory molecules such as CD244 and programmed death-1 (PD-1) on NK cells and T cells, as well as decreased serum cytotoxic effector molecules such as perforin and granzyme A, were noticed. Previous research has also found exhaustion phenotypes of CD8^+^ T cells in patients with severe COVID-19, which is caused by the upregulation of inhibitory receptors (IRs), which may impair host defences and lead to poor disease outcomes (Caruso et al., 2020; De Biasi et al., 2020; Diao et al., 2020; Laing et al., 2020; Schultheiß et al., 2020; Song et al., 2020; Zheng et al., 2020).

However, immune dysregulation may result in the COVID-19 severity, this functional exhaustion and subset alteration of NK and T cells may have a negative effect on efficient cell function during anti-tumor immune responses (McKinney and Smith, 2016; Wang et al., 2017; He et al., 2019).

Although the role of immune dysregulation and robust production of cytokines in COVID-19 pathogenesis has been well documented, future studies will be vital to investigate the shared molecular pathways and possible interaction between COVID-19 and the development and progression of cancer.

## Integration of SARS-CoV2 Genome and Cancer Induction; be or not to be

**FIGURE 3:**
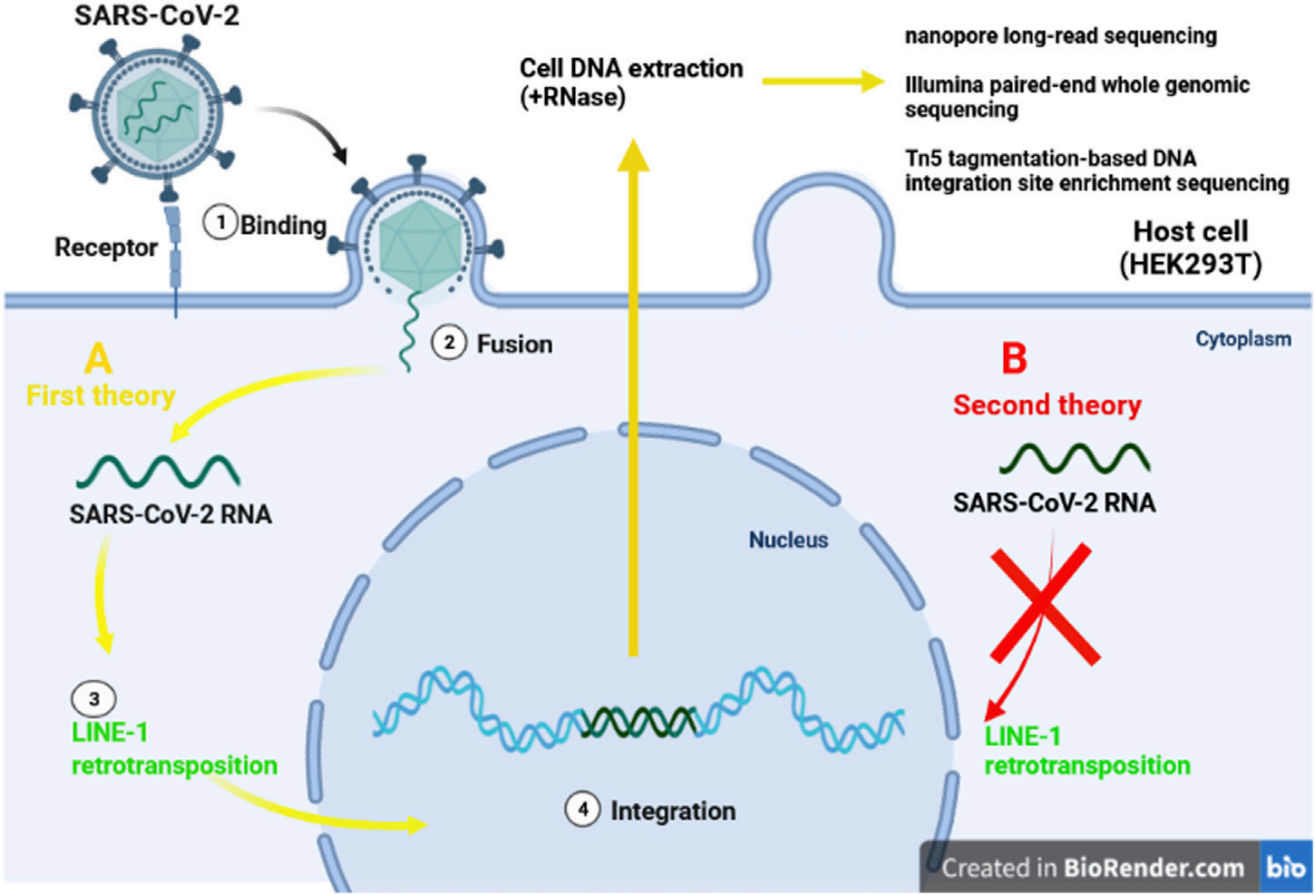
Two different theories of integration SARS-CoV-2 RNA. The first idea claims that LINE-1 retrotransposon proteins (A/yellow lines) can incorporate SARS-CoV-2 into human DNA. Despite the presence of the LINE-1 protein machinery (B/red line), the second explanation finds no evidence of virus genomic integration.

The World Health Organization, considers the infections as inducers for 15.4% of all cancers, with the viruses constitute up to 9.9% of all cancer causig infectious agents. The most prominent viral carcinogenic agent known to be hepatitis B virus, hepatitis C virus, human papillomavirus, and Epstein-Barr virus (Plummer et al., 2016). Integration of the viral genome into the host genome has been illustrated to be an unintended mechanism that may lead to the clonal growth and cancer develpemnt. In head-and-neck, cervical cancer, and liver cancer, the integration of viral genome into the host genom has been proven. Most of the geneome integrations were observed to be distributed across chromosomes, and a significant number of viral genome integrations occurred in the intronic (40%) regions, whereas only 3.4% of integrations were recognized in gene coding regions (Zapatka et al., 2020). In RNA viruses, the RNA genome is reverse-transcribed by the viral reverse transcriptase which subsequently integrates into the host chromosome shortly after infection, and expressed under the control of viral transcriptional regulatory elements. Following integration process, oncogenesis occurs due to activation or inactivation of the host genes, oncoproteins, and tumor suppressors (McLaughlin-Drubin and Munger, 2008). Several hypotheses have been put forward regarding integration of severe acute respiratory syndrome coronavirus-2 (SARS-CoV-2) into the human genome (Dai et al., 2020; Zhang et al., 2021) (Figure 3). Prolonged SARS-CoV-2 RNA shedding, and recurrence of PCR-positive tests, 60 days after the initial positive PCR test, have been widely demonstrated in non-infectious patients. These patients, howevere, do not appear to shed any infectious viruses (Bullard et al., 2020; Li et al., 2020d; Hosseini et al., 2020; Mina et al., 2020). Consequently, researchers have investigated the capability of SARS-CoV-2 as positive-stranded RNA viruses, for integration into human genome subsequent to reverse-transcription (RT). Hence, the transcription of the integrated sequences, provides logical evidence fo recurrence of PCR-positive tests (Dai et al., 2020). However, several investigations refuse the integration of SARS-CoV-2 into the human genome (Briggs et al., 2021; Kazachenka and Kassiotis, 2021; Parry et al., 2021; Smits et al., 2021).

To investigate the integration of SARS-CoV-2 RNA into the genome of infected human cells *in vitro,* Zhang et al. applied three different procedures: nanopore long-read sequencing, Illumina paired-end whole genomic sequencing, and Tn5 tagmentation-based DNA integration site enrichment sequencing (Zhang et al., 2020).

Recent studies provided enough evidences for the possibility of SARS-CoV2 integration into the human genome. The viral RNA found to be reverse transcribed in human cells by RT from LINE-1 elements or by HIV-1 RT. It has found the endogenous LINE-1 in human cells was induced to be overexpressed upon SARS-CoV-2 infection or following SARS CoV2-associated cytokine storm *in vitro*. The upregulation of host cell LINE-1 RT seems supportive for long-term positivity of PCR tests, even after recovery (Kazazian et al., 2017; Zhang et al., 2021). Although, Smits et al. applied long-read DNA sequencing to cultured HEK293T cells infected with SARS-CoV2 which provided no evidence for genomic integration of the SARS-CoV-2 into the host genome (Smits et al., 2021).

In another experiment, Briggs et al. examined 768 COVID patient’s nasal swabs in order to evaluate the positive role of viral genome integration on diagnostic PCR test for COVID-19. In this study, LINE-1-mediated retrotranscription of SARS-CoV2 RNA genome into the host DNA found to be a rare event with no practical impact on RT-PCR-based diagnostic capability. These data indicated the supposed SARS-CoV-2 integrations are likely artefactual, stemming from amplicon DNA contaminations or other unintended processes (Briggs et al., 2021).

As a consequence, the correlation between the integration of SARS- CoV-2 into the host genome, and cancer has remained unknown. Moreover, further studies on different species of SARS-CoV-2 are needed for more comprehensive conclusion.

## COVID-19 Diagnostic Testings and Risk of Cancer

In clinically COVID-19 suspected patients, computed tomography (CT) is the preferred imaging modality, and it is useful for monitoring patients during treatment. A dose reduction procedure is an essential requirement given the increased number of chest CT scans requested by referring physicians and the need to reduce the possible dangers posed to patients by ionizing radiation (Radpour et al., 2020; Azadbakht et al., 2021). Several works reported that the chest CT had higher sensitivity (around 97%) for COVID-19 pneumonia diagnosis compared to standard methods such as real-time PCR and NGS (Ai et al., 2020; Caruso et al., 2020; Fang et al., 2020). One of the major concerns is that CT ionising radiation may increase the risk of leukaemia and solid tumors incidence (Power et al., 2016). Some organ systems are highly radiosensitive, whereas others have stronger defences against the effects of ionizing radiation. Organs like the oesophagus, breast, and bladder, for example, are particularly vulnerable, although the rectum, pancreas, and prostate are far less so (Power et al., 2016). A study from February 24th to April 28th 2020 including 3,224 high-resolution thorax CT showed a very low risk estimation of cancer despite the impressive increment in thoracic CT examinations due to COVID-19 cases (Ghetti et al., 2020).

It has been suggested that one CT scan is equivalent to 300-400 chest X-rays, and that having multiple CT scans at a young age may raise the risk of cancer later in life. CT scans have two major dangers, according to the FDA. First, the test findings reveal a benign or coincidental finding, prompting unnecessary, perhaps invasive follow-up examinations that could pose further dangers. Second, x-ray radiation exposure increases the risk of cancer induction. The infection risk from contamination of surface, aerosolization during the CT acquisition of a Covid-19 patient, putting healthcare workers working in the CT suite at an increased risk of contracting this infection, and the CT imaging facility becoming a Covid-19 infection source to other patients undergoing CT examination are some of its limitations.

## Future Perspective

Future studies should focus on the disposition of patients recovering from the novel coronavirus for cancer. This increased risk to cancer could be related to the cumulative effect of different distinct aspects of coronavirus infection yet to be elucidated. Some of these aspects include the immune dysregulation, intensive production of cytokines, organ damage secondry to infection or other factors such as CT scans, and induced mutations as the result of the viral genome integration into the host genome.

While both innate and adaptive immune systems are indispensible to combat SARS-CoV-2 infection, uncontrolled host immune responses are among main COVID- related pathogenicity. Therefore, these two opposite aspects of the immune system should be addressed before putting forth fututre solutions to reduce post-infection side effects.

Future studies are vital to investigate shared molecular pathways and possible interactions between COVID-19 and the development and progression of cancer and to investigate whether the virus can be regarded as an etiological factor in the development of cancer. Our knowledge about coronavirus is relentlessly being updated and this review should be interpreted in light of future reliable findings.
